# Supplementary material for: Learning curve and surgical outcome of robotic assisted colorectal surgery with ERAS program
Source: Sci Rep. 2022 Nov 29;12:20566. doi: 10.1038/s41598-022-24665-w (PMC9709162; doi:10.1038/s41598-022-24665-w)
Supplement: Supplementary file 2 — Supplementary Information 2. [file 41598_2022_24665_MOESM2_ESM.zip › RAL Raw data-Ñ[▒K/099/OP_ñw╝╨░O▒Kñσ.pdf]

正式摘要(電子簽章：000000220947887)

請選擇...reporter

臺中榮民總醫院

Operation Note

[redacted]

- 手術日期：2021/07/17
- 手術醫師：VS 蔣鋒帆 , CR 林陳彥
- Anesthesia : GE
- Position : Lithotomy
- Aseptic agents : Betadine Aqua , Zephnan

Diagnosis

- Pre-operative diagnosis : Malignant tumor of rectum
- Post-operative diagnosis : s/p robotic low anterior resection

Surgical Indication

- For removing the primary colon tumor

Operation

- 1. Robotic (XI) assisted low anterior resection
  2. Intraoperative colonoscopy : (+)
  3. High ligation (+) : At IMA root (Via robotic clips). IMV was ligated via endoclip.
  4. Take down from the splenic flexure : (-)
  5. Robotic EndoGIA (45-4.3) X2 + EEA (29mm) were applied.
  6. Proximal cut end: > 10 cm, distal cut end: 3 cm
  7. No drainage tube was inserted
  8. ADHESION BARRIER FILM was placed

Finding

- 1. A 4 x 4 cm ulcerative tumor at upper rectum.
  2. No other gross tumor nor polyps was found
  3. There was no obvious visible liver metastasis, no visible peritoneal seeding
  4. No obvious ascites was noted
  5. Intra-operative colonscopy : The tumor location was confirmed, about 10~15cm FAV.
  6. Bilateral doughnuts : intact ; Air leak test: Done and no air leak was found

Procedure

- 1. General endotracheal anesthesia was induced and then the patient was placed in modified Trendelenburg lithotomy position.
  2. Preoperative antibiotics were given. A Foley catheter and nasogastric tube were placed.
  3. The abdomen was prepped and draped in standard sterile fashion.
  4. A small vertical incision was made above the umbilicus and a 8 mm trocar was inserted into the peritoneal cavity by open method. Peumoperitoneum was established with CO2 inflation.
  5. Laparoscopy was inserted and then other two 8mm trocars were inserted over LUQ, RLQ of abdomen. Another 12mm trocars was inserted over RLQ area, and the other assist-port 5mm troca was inserted on RUQ. The operative findings were mentioned as above.
  6. The sigmoid colon was held by grasper and the peritoneum overlying the mesentery was scored with electrocautery.
  7. The inferior mesentery artery was identified, ligated above the left colic artery with robotic clips, and transected. IMV was ligated via endoclip and then also transected.
  8. Dissection was done above the Gerota's fascia and toward the peritoneal attachments.
  9. The colon was mobilized from retroperitoneum overlying the sacral promontory and into the pelvis.

-
  10. Distal descending colon and s-colon were freely mobilized after the mesoclon was all dissected.
  11. The incision was then extended laterally and medially across the peritoneal reflection.
  12. The mesorectum was then mobilized using sharp dissection over the level of pelvic floor muscle. Then rectum was mobilized as possible. Vessel loops was applied over the rectum for better manipulation of the rectum.
  13. Colonscopy was done at the same time to eusure the margin of the desired resection point is adequate.
  14. The distal rectum was divided with robotic endoGIA (45-4.3) X2.
  15. The umbilicus area wound of abdomen was further extended. We approached into the peritoneal cavity by open method. S-colon and rectum was pulled out.
  16. The distal s-colon was then taken out for carefully inspection to make sure it has an excellent blood supply. The mesenteric fat was gently cleared from the terminal 2-3 mm of the bowel ends.
  17. The field was isolated with moist pad and the bowel opened and sized. It was determined that a 29 mm circular stapler could be accommodated. A purse string suture of 2-0 prolene was placed. The tumor-containing tissue was removed. Then the anvil was inserted, followed by the purse string suture. The colon was then put back to the abdominal cavity.
  18. The colon was then put back to the abdominal cavity and pneumoperitoneum was constructed again.
  19. The anal canal was irrigated with distil water and then the circular stapler was introduced through the anus and pierced through the upper area (The ventral site of the body) of the stapler line.
  20. Two complete donuts were obtained and the distal one was submitted to pathology for margin confirmation.
  21. The anastomosis was tested for leaks with irrigation solution and air introduced through the rectum and was found to be patent and intact.
  22. The bleeder was checked thorughly again.
  23. The 8mm trochar fascia wounds were all then closed with interrupted sutures via 1-O Vicryl.
  24. ADHESION BARRIER FILM was placed below the central abdominal wound.
  25. The extended fascia wound was closed with interrupted sutures via Num.1 Vicryl.
  26. Massive N/S was applied to irrigate the wound.
  27. The wound was further closed with 3-O Vicryl, and the skin was applied with SurgiSeal.
  28. The patient tolerated the procedure well and was brought to the POR in stable condition.
- Estimated blood loss: minimum
- 器械數量: 正確
- 紗布數量: 正確

Image

- PAINT
  - abdomen

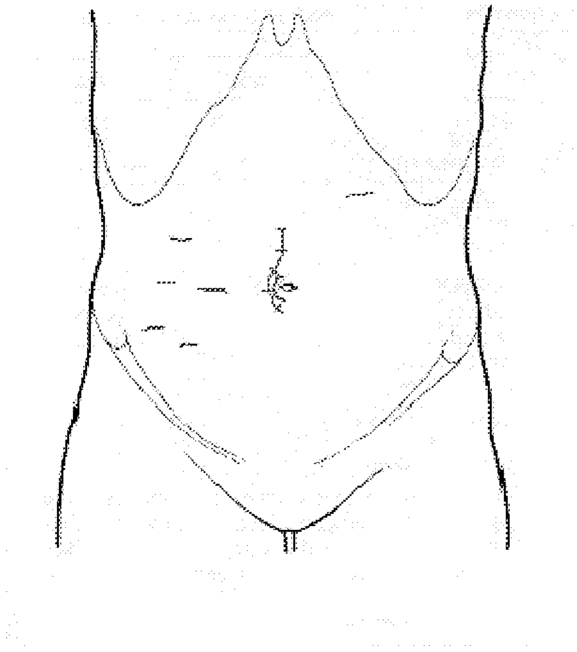

  - colon & rectum

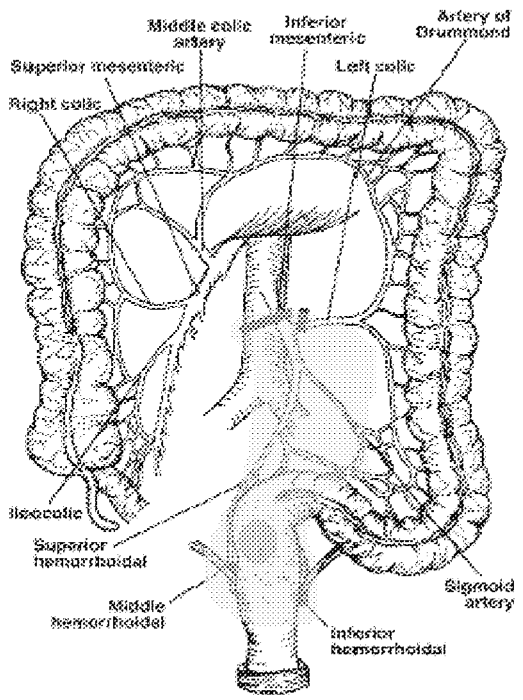

- The resected S-colon

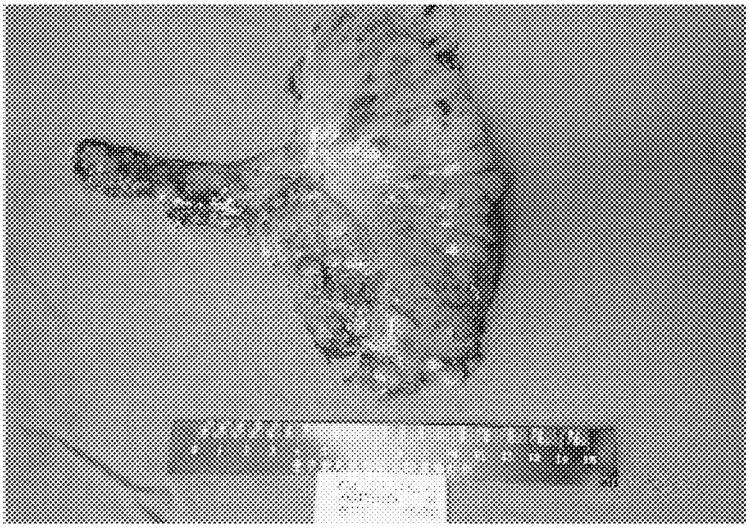

The ulcerative tumor

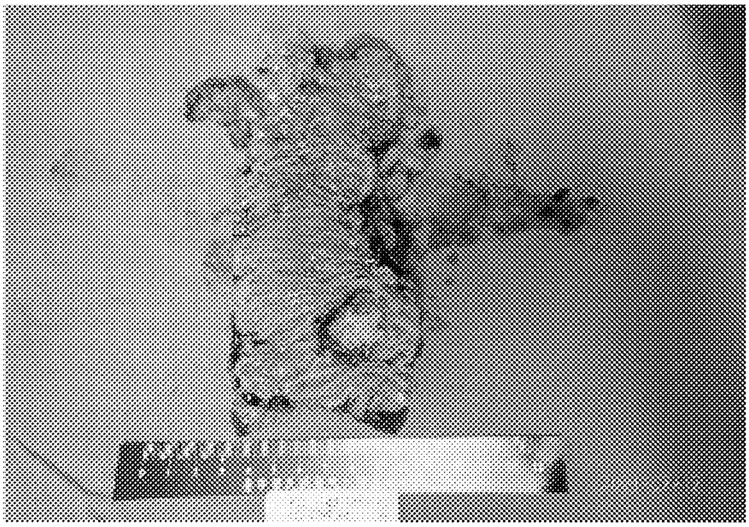

主任 2062F蔣鋒帆 / 醫師 2301H林陳彥

正式摘要(電子簽章：**000000220947887)**

請選擇...reporter

doctorRank:B
